# Dynamics of silver nanoparticle release from wound dressings revealed via in situ nanoscale imaging

**DOI:** 10.1007/s10856-014-5265-6

**Published:** 2014-07-11

**Authors:** R. David Holbrook, Konrad Rykaczewski, Matthew E. Staymates

**Affiliations:** 1 Nanomaterials Research Group, Materials Measurement Science Division, Material Measurement Laboratory, National Institute of Standards and Technology, 100 Bureau Drive, Gaithersburg, MD 20899 USA; 2 School for Engineering of Matter, Transport and Energy, Arizona State University, Tempe, AZ 85287 USA; 3 Surface and Trace Chemical Analysis Group, Materials Measurement Science Division, Material Measurement Laboratory, National Institute of Standards and Technology, 100 Bureau Drive, Gaithersburg, MD 20899 USA

## Abstract

The use of silver nanoparticles (AgNPs) in textiles for enhanced anti-microbial properties has led to concern about their release and impact on both human and environmental health. Here a novel method for in situ visualization of AgNP release from silver-impregnated wound dressings is introduced. By combining an environmental scanning electron microscope, a gaseous analytical detector and a peltier cooling stage, this technique provides near-instantaneous nanoscale characterization of interactions between individual water droplets and AgNPs. We show that dressings with different silver application methods have very distinct AgNP release dynamics. Specifically, water condensation on dressings with AgNP deposited directly on the fiber surface resulted in substantial and rapid AgNP release. By comparison, AgNP release from wound dressing with nanoparticles grown, not deposited, from the fiber surface was either much slower or negligible. Our methodology complements standard bulk techniques for studying of silver release from fabrics by providing dynamic nanoscale information about mechanisms governing AgNP release from individual fibers. Thus coupling these nano and macro-scale methods can provide insight into how the wound dressing fabrication could be engineered to optimize AgNP release for different applications.

## Introduction

Possessing strong biocidal properties, silver has long been used as an antimicrobial agent in wound care management [1, 2]. Medicinal use of silver began to decline, however, as antibiotics became more popular, powerful, and cost-effective. The widespread and often indiscriminant consumption of antibiotics has led to the emergence of antibiotic-resistant bacterial strains, which consequently has renewed interest in silver for medical applications [3, 4]. This interest, coupled with advances in the manufacturing of silver-incorporating products, has contributed to an increased use of silver-containing dressings [5–7]. Many dressings rely on silver nanoparticles (AgNPs) for efficient production of the toxic silver ion. Given their high surface area-to-volume ratio, AgNPs are a nearly-ideal ion delivery vehicle [8]. The use of silver-containing dressings in clinical settings, however, remains somewhat contentious as results of various efficacies have been reported [9–11].

The conflicting evidence regarding silver-containing dressings is caused by several factors, including: distinct silver delivery systems [5]; lack of standardized testing procedures that mimic real-world usage conditions [2, 12]; and unknown factors influencing silver (as both ionic silver and AgNPs) release rates [13]. In fact, total silver content, which is often used as a proxy of antimicrobial action, is only one of the important parameters governing a product’s efficacy [13–15]. According to these authors, other key parameters for effective antimicrobial ability in dressings include the distribution, chemical and physical form of silver, and absorbed moisture content. In other words, the silver delivery system, or the ability of a dressing to release sufficient quantities of silver in the proper form in an appropriate time frame, is the critical antimicrobial component. Consequently, a better understanding the antimicrobial mechanisms of silver as well as a thorough understanding of silver release kinetics could be used to substantially improve silver-impregnated products.

The antimicrobial potency of AgNPs is governed by two distinct mechanisms. The first is related to the ionic silver production via metallic silver dissolution. Ionic silver can react with the sulfhydryl groups of proteins, thereby disrupting the proton motive force and causing massive cation leakage (namely K^+^ and H^+^) [16] as well as disruption in cellular respiration and growth [17]. This mechanism, relating to the ionic fraction of AgNPs, has been called the “long-distance” effect [18] since ionic silver is more mobile than either dispersed or surface-embedded AgNPs. Assuming sufficiently high ionic silver concentrations, this “long-distance” effect would be lethal for most bacterial strains without sufficient silver-resistance means [19]. The second mechanism is the so-called “short-distance” effect and is related to the direct interaction between the cell and the AgNPs [18]. In this scenario, AgNPs could cause a nanomechanical disruption of the cell membrane [18], locally deliver high intracellular concentrations of ionic silver [20], and/or cause oxidative damage via reactive oxygen species production [21]. This “short-distance” toxicity may be influenced by bacterial type [18], environmental conditions [22], and AgNP diameter [23] and is therefore more difficult to evaluate compared to ionic silver toxicity. The direct AgNP toxicity, however, may be most beneficial against silver-resistant strains [19] or antibiotic-resistant biofilms [24]. Since neither silver ions nor silver nanoparticles offer robust coverage by themselves, an ideal silver-delivery system for wound care dressings would contain both “long-” and “short-distance” components.

Meaningful strategies to evaluate these two components under realistic conditions, especially the direct, “short-distance” AgNP portion, are lacking. Silver release investigations involving wound dressings typically utilize full saturation of the tested fabric for long periods [5], which would promote ionic silver release and, concomitantly facilitate AgNP transformation such as dissolution, agglomeration and aggregation [25]. Such transformation would not accurately characterize AgNP release when AgNP-impregnated dressings are directly adjacent to wounds. Further, not all wounds, involve moisture levels comparable to these test conditions. To our knowledge, investigation into mechanisms of rapid release of AgNPs from wound dressings upon water contact has not yet been investigated. AgNPs immediately released, however, is likely to be most representative of materials that the dermal layer would encounter with AgNP wound dressings.

The objective of this work is to conduct real-time, in situ nanoscale characterization of AgNPs being released from silver-impregnated dressings during simulated wetting/drying cycles. This type of investigation can provide insight into the dynamics of the “short-distance” mechanism of antimicrobial potency of AgNPs. To achieve this, we utilize a combination of an environmental scanning electron microscope (ESEM), a gaseous analytical detector (GAD) and a peltier cooling stage. Using this technique, we study behavior of AgNP during water condensation and evaporation on wound dressing barriers with AgNP either deposited on or grown from the fiber surface. We clearly observe distinct AgNP release mechanisms during wetting of the two dressings, demonstrating the use of ESEM-GAS-EDS to characterize the released form of the solid material prior to any major physical transformation.

## Materials and methods

### Wound dressings

Two commercially available silver-impregnated wound dressings were purchased and labeled dressing 1 and 2. These dressings were chosen for their relatively high yet comparable silver content (approx. 108,500 mg Ag/kg material). Extensive characterization on these and other commercially available nano-based textiles has been reported elsewhere [26].

### Sample preparation, equipment, and operation

An ESEM (Quanta 200 FEG ESEM) and gaseous analytical detector (GAS) (FEI, Hillsboro, OR) combined with an energy dispersive X-ray spectrometer (EDS) (Bruker, Ewing, NJ) was used for imaging, particle size distribution measurements, and determination of particle composition, respectively. The ESEM has a differential pumping system that enables high resolution imaging while the microscope chamber is filled with water vapor at pressures up to 2.7 kPa. Since the water triple point occurs at 0 °C and 610 Pa, decreasing sample temperature using the peltier cooling stage enables water to condense on the sample substrate. ESEM has been previously used to visualize water micro-drops on fibers and hydrophobic nanoparticle motion on liquid–vapor interfaces [27–30]. We adopt this technique to image nanoparticles on water condensed on and around the dressing fibers. The GAS has a 8.5 mm long cone that extends down from the detector and facilitates EDS analysis at low vacuum conditions [31]. This detector collects high energy backscattered electrons, which provides higher sensitivity to subsurface features than secondary electron imaging for electron-dense materials. Small swatches (5 mm × 5 mm) were cut from each dressing using polytetrafluoroethylene scissors. These swatches were attached to double sided copper tape, which was then placed on a custom-built peltier cooling stage. To optimize the temperature control, the sample was directly attached to a 1.2 cm × 1.2 cm Thermoelectric Cooler (TEC) module from Analog Technologies (Burnsville, MN) mounted on a copper block cooled to 4 °C with a constant flow of chilled water. To reduce the thermal interface resistance, vacuum compatible thermal grease (Apiezon-N from SPI, Landover, MD) was applied to the TEC module and copper block interface. A LM35 Precision Centigrade Temperature Sensor (Texas Instruments, Dallas, TX) was used to measure the surface temperature of the TEC. The TEC and the LM35 sensor were mechanically clamped to the cooling block. Further details of the custom-built peltier cooling stage are found elsewhere [32]. After sample loading and two standard purging cycles in the ESEM, the pressure was maintained between 900 and 950 Pa. Water condensation on the dressing was facilitated by decreasing the temperature of the TEC between 2 and 4 °C at a constant chamber pressure. Individual fibers in close contact with the copper tape were most suitable for assessing AgNP release.

Experiments with each fiber were conducted on two separate occasions and images were collected from multiple locations (n > 10) within the same fiber swatch.

### Image collection and processing

All images were collected at 20 kV, and a working distance of 10 mm. The dynamics of the wetting process were imaged with a dwell time of 1–3 µs per pixel and (512 × 471) pixel or (1,024 × 943) pixel frame sizes. The corresponding images were saved every 0.2 or 1 s. The images were analysed using ImageJ [33]. EDS Identification of individual nanoparticles was achieved with the electron beam in “spot mode”. Particle size distribution (PSD) measurements were made with Image-Pro (Media Cybernetics, Rockville, MD) using multiple, high magnification ESEM images. Not all the measured AgNPs were spherical and were therefore reported as “equivalent nanoparticle diameters”. At least 100 individual AgNPs were measured for each PSD.

## Results

**Fig. 1:**
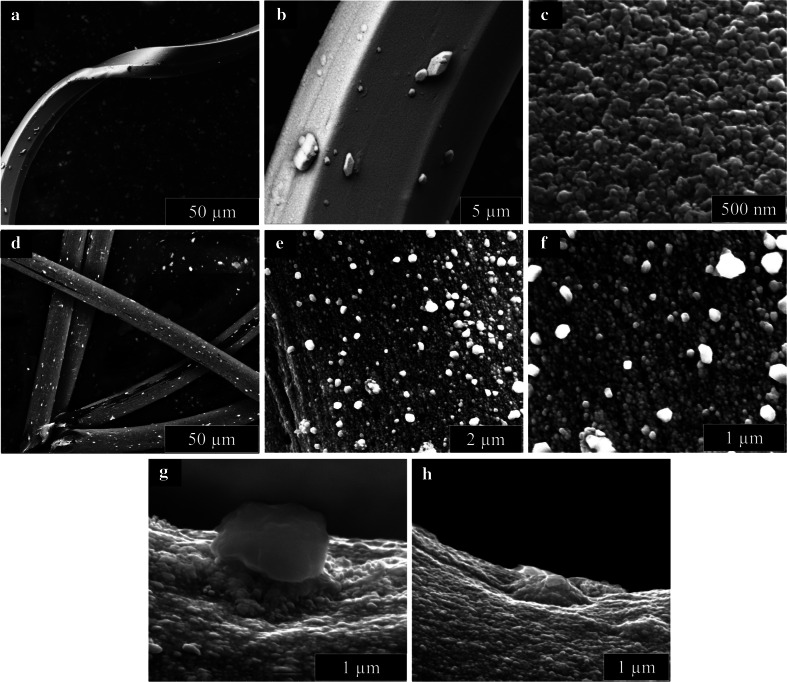
ESEM images of **a**–**c** dressing 1 and **d**–**h** dressing 2 at varied magnifications; **g**, **h** demonstrate varied morphology of larger AgNP aggregates from dressing 2

**Fig. 2:**
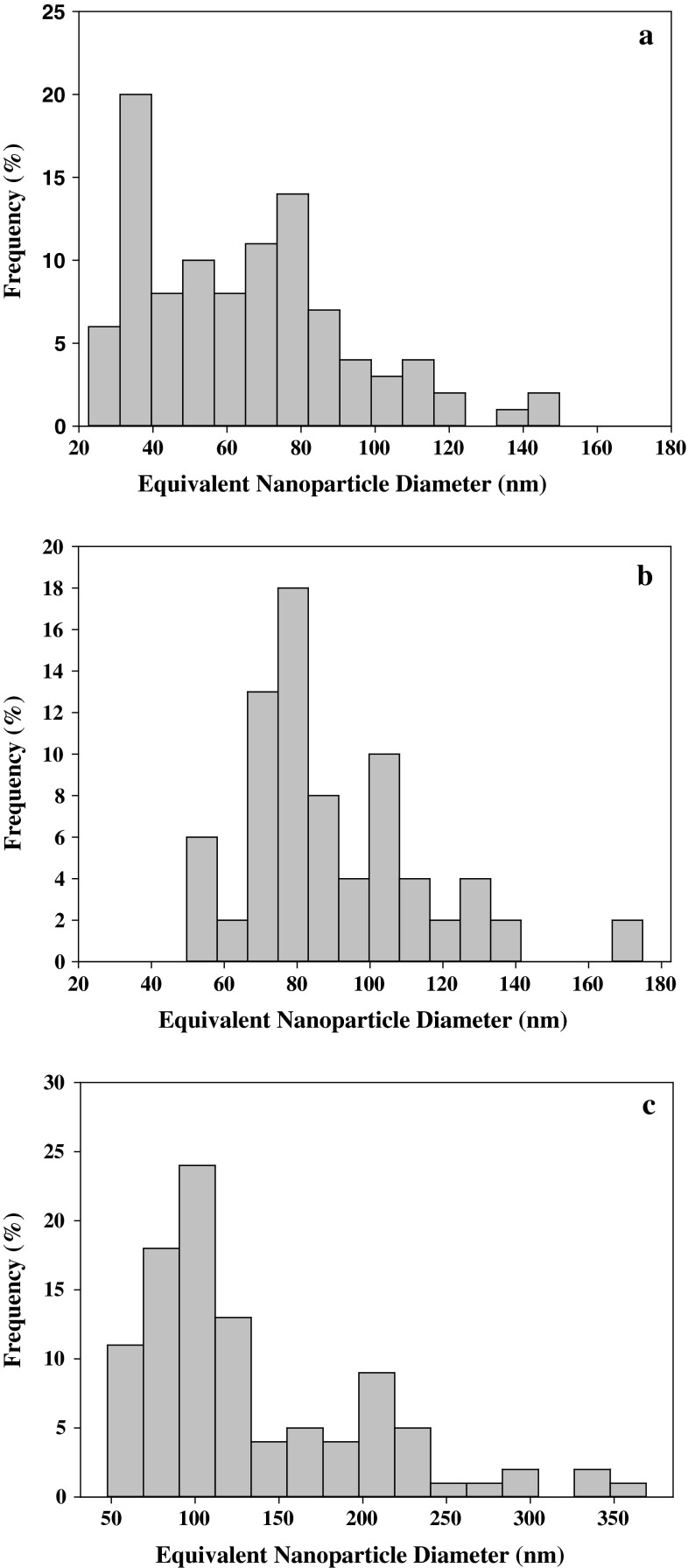
Particle size distribution histograms for **a** AgNPs on surface of dressing 1, **b** AgNPs in solution that were released from dressing 1, **c** AgNPs on surface of dressing 2. There was insufficient AgNP release to construct a representative AgNP PSD for dressing 2

We study two commercially available dressings for wound care purposes which, based on electron microscopy characterization, appear to have similar silver delivery vehicles in that both are covered by a conformal silver film with distinct AgNP clusters (Fig. 1a–f). There are, however, differences in the AgNP morphology and PSD of the two dressings. The AgNP film on dressing 1 consists of larger aggregates made from distinct smaller AgNPs. There is more space between adjacent aggregates compared to adjacent small AgNPs within the aggregates, although both contribute to a fine surface roughness that is apparent at high magnification (Fig. 1c). The surface roughness of dressing 2 is apparent at lower magnifications and is attributed to the large, individual AgNPs located at the surface of the dressing (Fig. 1d, e). These larger AgNPs appear slightly more spherical than the smaller, individual AgNP, which are observed at higher magnification (Fig. 1f). There appear to be two distinct populations of the larger AgNPs on dressing 2. When viewed laterally, some of the larger AgNPs stand on top of a pile of smaller AgNPs and are located away from the dressing surface (Fig. 1g), while the second population appears more entrenched and is directly adjacent to the surface (Fig. 1h). Further, while both PSDs are fairly broad, spanning sub-20–160 nm for dressing 1 and 50–375 nm for dressing 2 (Fig. 2a, c, respectively), the AgNPs on dressing 1 has a significantly smaller average equivalent diameter (64 ± 28 nm, average ± standard deviation, n = 110) compared to dressing 2 (135 ± 71 nm, n = 112).

**Fig. 3:**
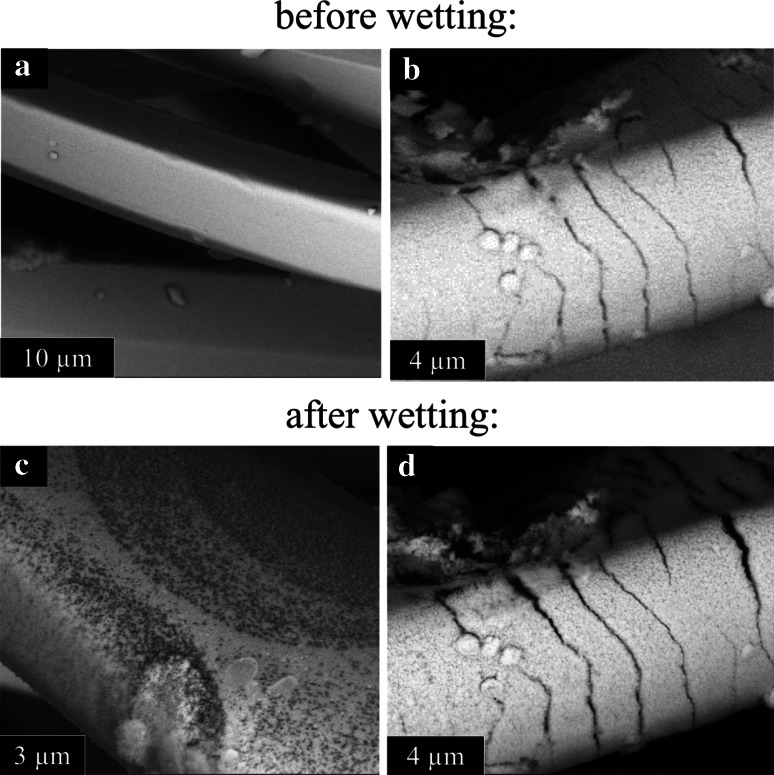
ESEM images of AgNP on dressing 1 fabric before (**a**, **b**) and after (**c**, **d**) fiber wetting

**Fig. 4:**
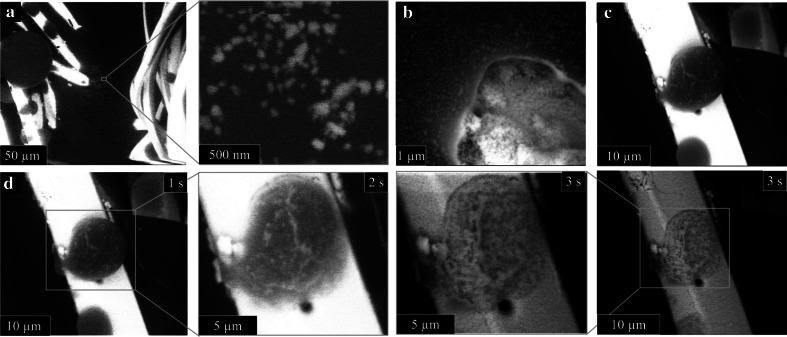
In situ ESEM images of dressing 1 wetting process showing AgNPs released into **a** water layer near the fibers, **b** in the meniscus wetting the fiber, and **c** on individual condensed drops on the fiber; **d** forced electron beam induced evaporation of water micro-drop shown in **c** demonstrating AgNP redeposition on the fiber. The identity of the AgNP ensembles was in situ verified via EDS

The real-time, in situ images captured with this instrumentation were critical in characterizing the AgNP behavior during the condensation/evaporation process, both during and immediately following wetting of the dressing. Prior to wetting, the silver film on the surface of dressing 1 appears largely intact (Fig. 3a), although cracks in this film are apparent and may have been introduced via mechanical stresses during dressing handling (Fig. 3b). Once dressing 1 is contacted with water, rapid and extensive detachment of AgNPs from the fabric occurs. For example, dense populations of individually-resolved, floating AgNPs within a 50 µm radius of the fiber-water interface can be seen (Fig. 4a). This population begins to grow as the water travels along the fiber surface (Fig. 4b) and remain relatively close (within a few hundred nanometers) to the fiber surface. The very close proximity between the AgNPs and the fibers suggests the AgNPs have likely originated from the individual fibers. This premise was further supported by observing AgNPs within a drop that was condensed on a single fiber (Fig. 4c). The AgNPs observed within this drop must have been released during the micro-droplet growth process since the drop was physically separated from other areas of the dressing, thereby eliminating any potential longer range AgNP transport.

Depending on the severity of wetting, the released AgNPs can be redeposited on the fiber surface (Fig. 4d) or washed away from the fiber to collect on the copper substrate (data not shown). To illustrate this point, we forced micro-droplet evaporation with electron beam heating achieved through imaging area reduction [34, 35]. This rapid evaporation of the micro-droplet causes re-deposition of the suspended AgNPs, resulting in an obvious rearrangement of AgNPs on the fiber surface (Fig. 4d). Larger micro-droplets can cause substantial silver film etching, where the underlying supportive structure is nearly visible following prolonged wetting (Fig. 3c). Furthermore, the cracks in the silver film that may have been introduced during sample handling have widened following wetting (Fig. 3d), suggesting that such surface defects may contribute to, but are not necessary for, AgNP release during wetting. Consequently, wetting and drying of dressing 1 will lead either to AgNP rearrangement on the fiber surface and/or a removal of AgNPs from the fiber surface to the surrounding environment.

The image sequence in Fig. 4 shows that AgNPs released from dressing 1 remain suspended in the water droplets throughout the experimental period. The PSD of the released AgNPs was shifted to a slightly larger population when compared to the pre-wetted surface (Fig. 2b), althought the average equivalent diameter of the released AgNP (89 ± 26 nm, n = 134) was not significantly different.

**Fig. 5:**
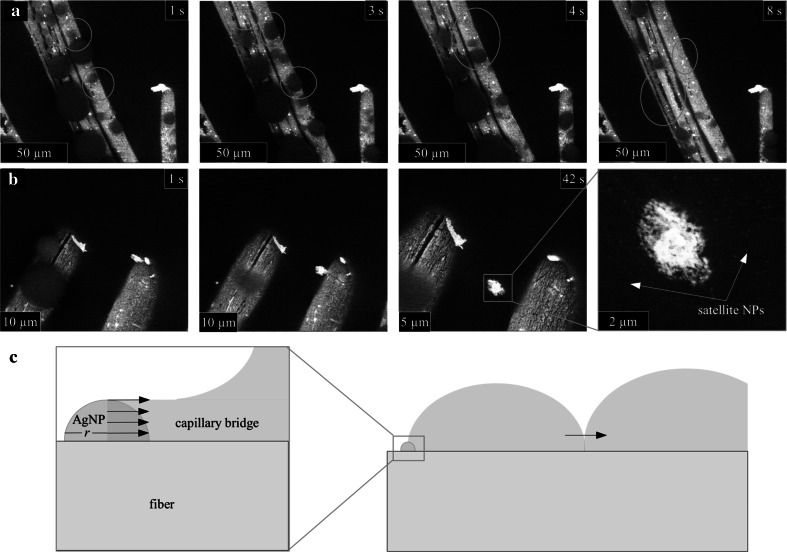
In situ ESEM images of dressing 2 wetting process showing **a** multiple water micro-droplet growth and coalescence cycles (with one another as well as with the water surrounding the fiber) without large nanoparticle release and **b** slow release of a large nanoparticle showing multiple satellite nanoparticles surrounding the original particle after its release; and **c** schematic of the simplified model of forces induced on AgNP by solid–liquid–vapor triple line while emerging from a coalescing micro-droplet

In contrast to the rapid AgNP release observed in dressing 1, AgNP release from dressing 2 was more limited. Despite being subjected to repeated condensation and evaporation cycles (resulting in wetting and drying in the same local area), substantial AgNP release was not detected (Fig. 5a). We did, however, observe the occasional release of larger AgNP aggregates following prolonged soaking in water (Fig. 5b). Additionally, very small (~5 to 10 nm) high-contrast particles were also observed floating near the released aggregate (Fig. 5b). These nanoparticles may be single AgNPs released between the aggregate and fiber surface or from disaggregation of the original large AgNP aggregate. While EDS could not directly confirm the composition of these particles due to the small size and low packing on the liquid surface, they are likely AgNPs since large area EDS analysis did not identify any other metallic species present on the fibers. There was insufficient release to construct a representative AgNP size distribution histogram.

## Discussion

The observed differences in AgNP morphology, PSD, and release behavior between the two dressings suggest that two distinct processes were used for silver application. In general, there are three methods for adding silver to textiles: embedding silver additives within a synthetic fiber polymer; incorporating silver additives into a polymer solution, which is then used to coat the fiber surface; and either forming or directly adding AgNPs directly to the fiber surface [36]. AgNPs were incorporated into dressing 2 using electroless silver plating, where AgNPs are grown at the polymer surface. Although the manufacturing process for the dressing consisting of dressing 1 is patented, based on the results presented above, we propose that a direct AgNP deposition method is used. Characterization of consumer products that incorporate engineered nanomaterials is typically limited to, for example, the physical and chemical nature of the specific nanomaterial, including size, shape, distribution, elemental composition and quantity. While this type of information is helpful, even critical, in cataloging nano-based consumer products, the results of the current work highlight the need for a more thorough understanding of nanomaterial behavior when using such products [37]. Standard protocols used for dressing characterization do not capture the observed differences in AgNP release. Indeed, total silver release from wound dressings is related to the amount [38] and chemical composition [5] of the adsorbed moisture and not to total silver content [13]. These findings indicate that a more comprehensive characterization paradigm is necessary to better inform product users of nanomaterial behavior.

The higher frequency of larger AgNPs particles released from dressing 1 compared to the PSD of the original material (Fig. 2a, b) suggests two plausible mechanisms to explain the observed behavior. The first mechanism is that AgNPs are not released from the dressing as individual particles but as small aggregates. While small aggregate release was not directly observed, adhesion between adjacent AgNPs could be greater than between the AgNP and the fiber surface. The second potential mechanism is that individual AgNPs are, in fact, released but are colloidally unstable and quickly agglomerate/aggregate in solution. Partial agglomeration/aggregation of individual AgNPs in the water was directly observed, especially during water droplet transport. Another process that can impact PSD measurements is AgNP dissolution (i.e., towards smaller particles). No definitive conclusions, however, can be made regarding dissolution of attached or suspended individual AgNPs. Dissolution occurs in the first few surface monolayers of the AgNPs [39, 40] and is therefore beyond the resolution of the ESEM under the conditions used in this investigation [31]. Both AgNP dissolution and agglomeration/aggregation would be expected during periods of prolonged wetting [41].

The current work suggests that ionic silver and AgNP release dynamics occurs by different mechanisms. While ionic silver release is largely governed by dissolution [40, 42], AgNP release can be caused by the shear stress induced by deformation of the solid–liquid–vapor triple line during micro-droplet movement. As illustrated in Fig. 5c, this process occurs when a AgNP submerged within a micro-drop is exposed due to the drop motion, and is analogous to triple line pinning around sparse defects, which can cause strong drop contact angle hysteresis [43, 44]. These particles act as pinning sites, creating capillary bridges that deform the triple line [45]. With assumption of nearly perfect wetting of the AgNP, the force exerted by the liquid bridge on a hypothetical semi-spherical nanoparticle can be approximated as the product of water surface tension, σ, and the wetted perimeter of the AgNP with radius *r*, equal to *π r*. Therefore, the shear stress on the nanoparticle–fiber interface scales with the ratio of σ/*r*. According to this scaling, the shear stress experienced by a 20 nm diameter AgNP is 100 times greater than that experienced by a 2 µm diameter AgNP aggregate. As a result, smaller, individual AgNPs would be subjected to much higher shear forces than larger AgNP aggregates during micro-droplet movement.

This explanation is consistent with the observed AgNP release dynamics for dressing 1, where more smaller AgNPs were released (and subsequently aggregated/agglomerated) compared to the larger aggregates (Fig. 2a, b). Dressing 2, by comparison, used electroless silver plating to produce AgNPs that extended above but are anchored to the supporting polymer matrix. The resulting shear force from droplet movement is not sufficient to overcome the anchoring force and, consequently, very few AgNPs are released. The release of larger AgNP aggregates that were released during the in situ wetting experiment may be attributed to the differences in their morphology. In particular, we suspect that the larger AgNPs resting on top of a smaller AgNPs pile (Fig. 1g) are released preferentially as compared to the more “embedded” AgNP aggregate (Fig. 1h) due to combination of small AgNP “neck” erosion via dissolution and shear stress. The “neck” erosion mechanism also explains the presence of individual satellite AgNPs observed during the large AgNP aggregate release (Fig. 5b).

The images obtained in these dynamic experiments provide an insight into the real-time behavior of AgNPs immediately following dressing wetting and lead to three important findings regarding AgNP behavior. First, whereas ionic silver release from dressings is related to the solution’s ionic content [46], the AgNP release dynamics are dependent upon fiber-liquid interactions (including wicking and wetting) [47]. Potentially, fiber wetting properties could be manipulated to release optimized quantities of AgNPs and therefore be considered a parameter when making dressing selection and design [13]. Second, smaller AgNPs (<100 nm) are rapidly released upon water contact. This observation has important implications for AgNP transport during open wound care, because patients may unwittingly be exposed to high concentrations of AgNPs; patient sensitivity to silver may be an important consideration [48]. Third, short wetting/drying cycles can result in substantial changes to the AgNP surface distribution and migration into the environment. The impact that AgNP rearrangement has on, for example, anti-microbial properties are currently unclear but there are striking differences between a dressing 1 that has undergone a single wetting/drying cycle and those that have not (Figs. 2c, 4d). Last, it is currently unclear what influence, if any, the biomolecules contained in complex biological fluids has fiber–liquid interactions and AgNP release dynamics [49, 50]. Incorporating these molecules into future experiments may provide a greater confidence in AgNP behavior during patient care.

## Conclusions

We have demonstrated that the ESEM-GAS-EDS method can be used for in situ characterization of AgNP release processes from wound dressings. The environmental conditions maintained in the current work (small droplet size, comparatively rapid condensation/evaporation cycle, non-saturation) approximate real-world conditions wound dressings typically encounter. In this sense, the use of ESEM-GAS-EDS can help to characterize immediately the form of the solid material release (i.e., nanoparticle or particulate) prior to any major transformation, thereby providing critical information on AgNP exposures during product use. The silver surface coating procedure and the fiber-liquid interaction appear to control the AgNP release dynamics. Both parameters could be exploited for better control over silver release such that dressing can be optimized on a case-by-case basis.
